# Supplementary material for: VEGF-A, PDGF-BB and HB-EGF engineered for promiscuous super affinity to the extracellular matrix improve wound healing in a model of type 1 diabetes
Source: NPJ Regen Med. 2021 Nov 18;6:76. doi: 10.1038/s41536-021-00189-1 (PMC8602425; doi:10.1038/s41536-021-00189-1)
Supplement: Supplementary file 1 — Supplementary information. [file 41536_2021_189_MOESM1_ESM.pdf]

Supplemental figures and legends:

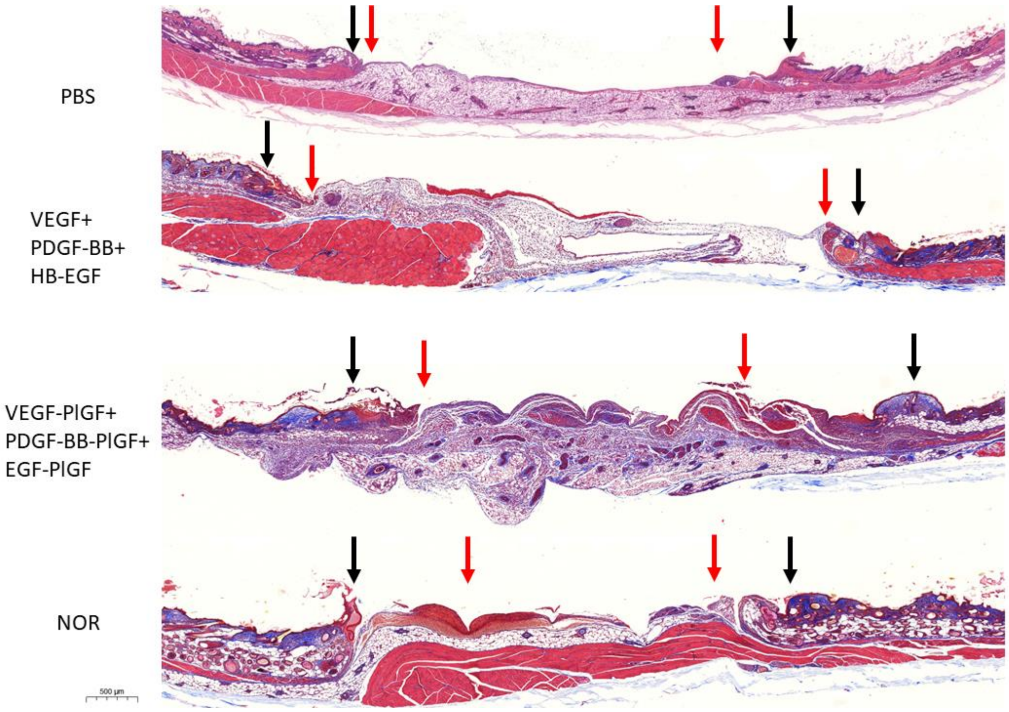

Supplemental figure 1: The triple therapy of VEGF-PlGF-2$_{123-144}$, PDGF-BB-PlGF-2$_{123-144}$, and HB-EGF-PlGF-2$_{123-144}$ does not cause increased scar tissue in the epithelium. Wounds were treated with the indicated growth factors (or PBS) and allowed to heal for 7 days, sectioned and stained using Masson's trichrome in addition to Hematoxylin and Eosin (Fig 1E). No scar tissue was observed within the healing wounds. Representative images, size bar = 500 µm.

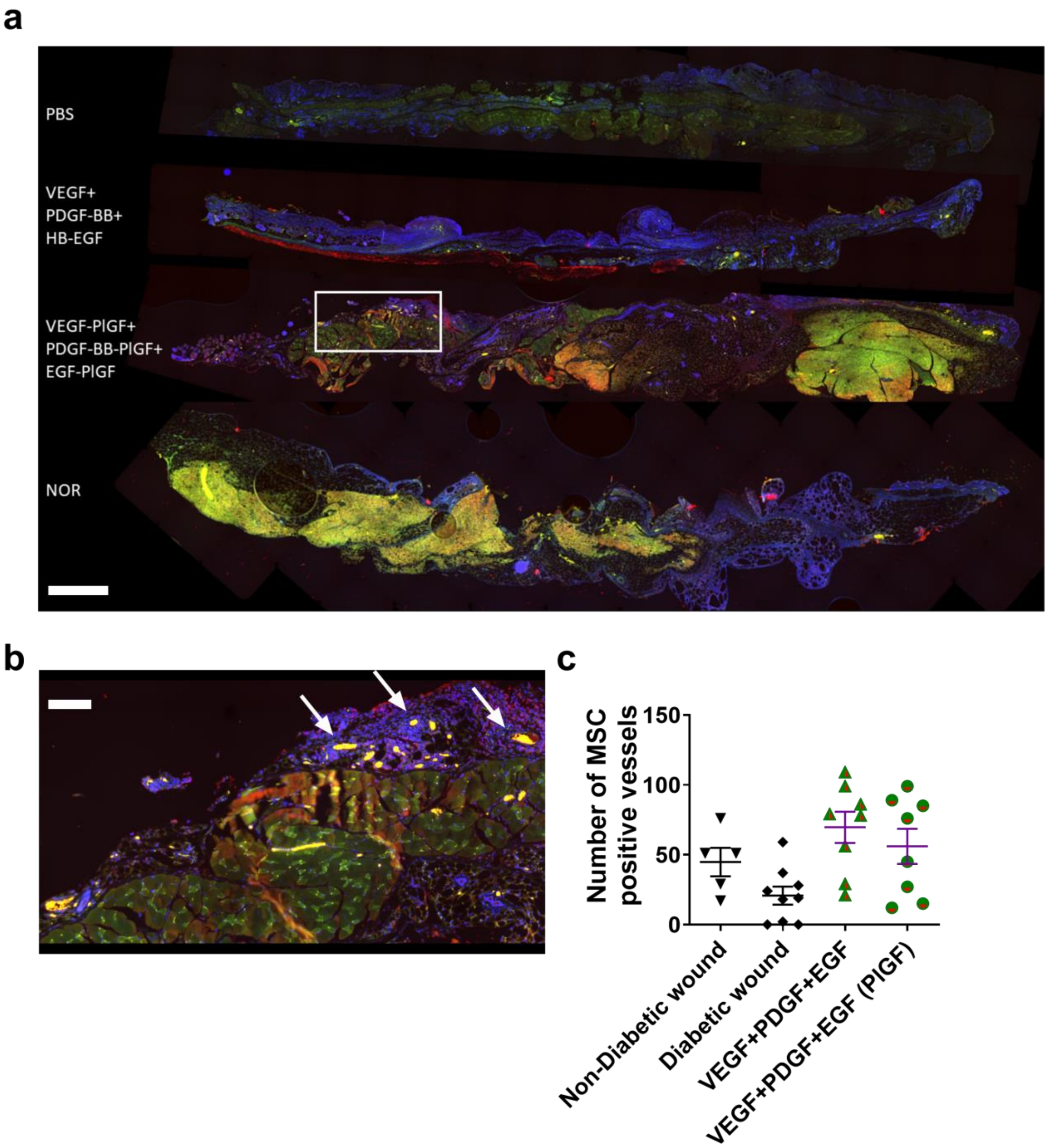

Supplemental figure 2, MSC staining in wounds. (A) Wounds with 7 days of healing were sectioned and stained for MSC using DAPI, CD90 (488), CD73 (647). (B) inset of A. Arrows indicate blood vessels for the purpose of counting, data in (C). There is no statistical significance in (C) by ANOVA or Student's t-test. Red = CD73, green = CD90, blue = DAPI. Size bar = 1 mm, inset size bar = 100 µm. Error bars are SEM.

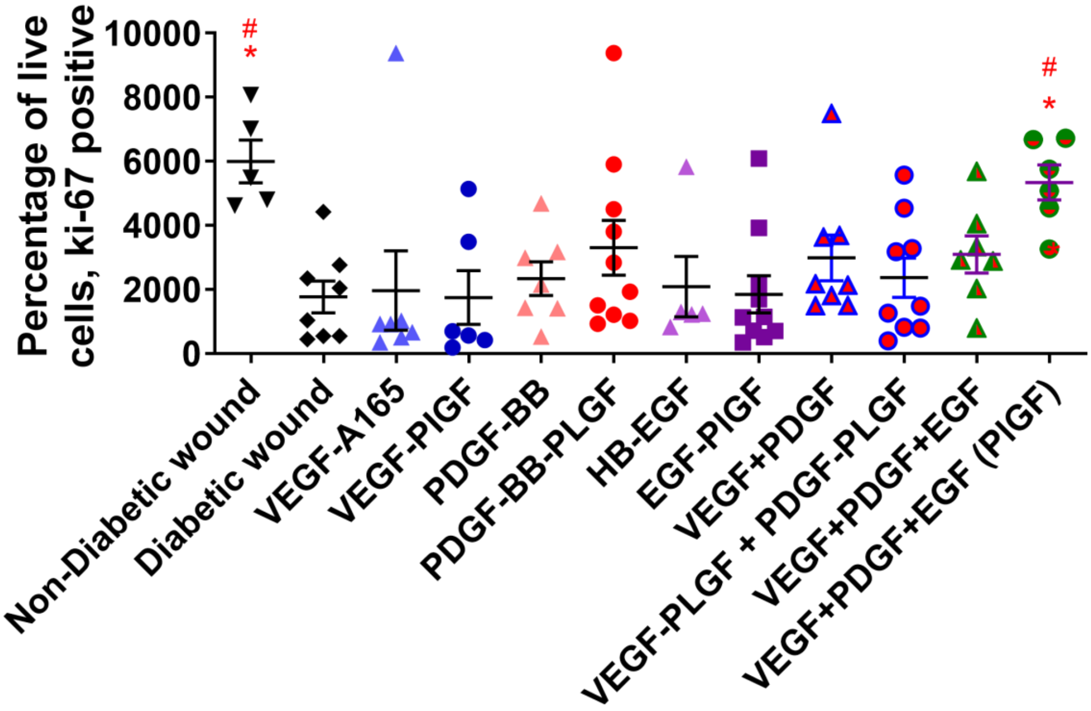

Supplemental Figure 3. Treatment with triple therapy (VEGF-PlGF-$2_{123\text{-}144}$, PDGF-BB-PlGF-$2_{123\text{-}144}$, and EGF-PlGF-$2_{123\text{-}144}$) increases the number of proliferating cells in wounds. Proliferating cells (ki-67) at 7 days healing. N ranges from 5 to 10. * denotes comparison to the diabetic wound. # denotes a comparison between untreated NOD wound and untreated NOR wound, and the WT GF(s) are compared to their counterpart -PLGF-$2_{123\text{-}144}$ variant(s), e.g., VEGF vs VEGF- PlGF-$2_{123\text{-}144}$. * = $P < 0.05$, $P < 0.01$, $P < 0.001$, 1 way ANOVA. # = $P < 0.05$, $P < 0.01$, $P < 0.001$, Student's t-test. Error bars are SEM.

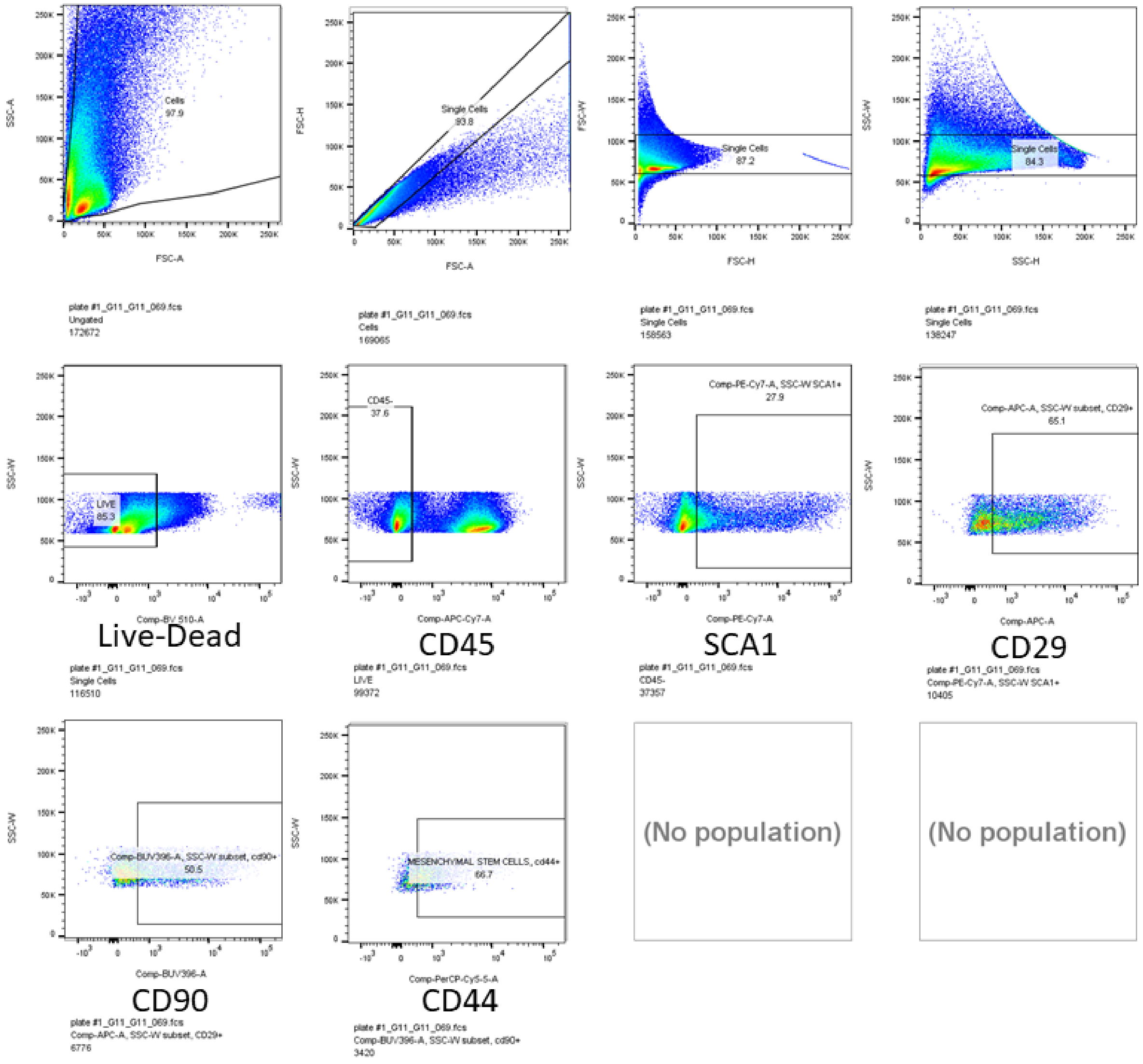

Supplemental Figure 4. Gating strategy for MSC. Cells were gated for live cells using FSC-a vs SSC-a, followed by FCS-a vs FSC-h, followed by FSC-h vs FSC-w, followed by SSC-h vs SSC-w, then followed by live-dead aqua staining. We could expect 10,000+ cells per wound aliquot.

Supplemental statistics note

This study was designed to answer two questions. First, whether the eGF triple therapy treatment (VEGF-A-PlGF-$2_{123\text{-}144}$, PDGF-BB-PlGF-$2_{123\text{-}144}$, and EGF-PlGF-$2_{123\text{-}144}$) improves wound healing for mice with T1D in a statistically significant fashion, and second, whether changes in the cellular milieu of a wound affects healing.

We answered these two questions using two different response variables: the amount of granulation tissue (measured in mm^2) and the wound extent, i.e., the diameter of the wound (measured in mm) at its widest point. As granulation tissue is related to the thickness of the wound, while wound extent is related to the wideness of the wound, these two response variables are not directly related to one-another.

Importantly, it is possible for an explanatory variable to be linearly related with both of them.

We ran a large number of experiments, featuring different treatments (always including control mice) and measuring properties of the mice (age, etc.) as well as numbers of cells of different types. In each experiment, initial wounds were circular, with a diameter of 6 mm. Our experimental protocol followed the "walk-in clinic" model where mice would become diabetic spontaneously and only then be suitable to be included in an experiment. Consequently, different experiments had different numbers of mice, generally around 6 or 7 per experiment. Our statistics regarding the control group are aggregated from all experiments, as control mice always had the same experience. Hence, the control group contains 20 mice.

To answer whether the triple therapy treatment has a statistically significant effect on wound healing for mice with type I diabetes, we considered ten different treatment options, as well as a group of diabetic mice receiving no treatment, and a group of non-diabetic mice receiving no treatment. For each of the twelve groups, seven mice received the treatment and we measured the two response variables

after seven days. We used an ANOVA test to compare these ten groups (Kruskal-Wallis for granulation tissue) and found that there was a statistically significant difference between them. We then conducted post-hoc tests (using Tukey's HSD procedure) to compare each treatment to the untreated diabetic wound group.

As illustrated in Figure 1, several treatments were statistically significantly more effective than no treatment, with the largest impact being the triple therapy treatment. Furthermore, as expected, there was a statistically significant difference between the diabetic no-treatment group and the non-diabetic no-treatment group.

Next, we considered whether or not these differences were visible already on day three of the wound, rather than day seven. In this case, each group had n = 3 mice (so as to avoid giving an overly large number of mice treatments known to be suboptimal), though the triple therapy groups (-PlGF-$2_{123\text{-}144}$ and WT) had n = 7 in each group, for reasons we will shortly explain. The result, shown in Figure 1, is that again the triple therapy was statistically significantly better than no treatment, for the response variable of wound extent (again using an ANOVA/Kruskal-Wallis test with appropriate post-hoc tests). We note that three days was not enough to see the difference for the response variable of wound area.

Next, we considered whether or not the addition of the -PlGF-$2_{123\text{-}144}$ peptide to the GF (resulting in eGFs) was significant. Again, at both three days and seven days, we used Student's t-tests (or Mann-Whitney tests) to compare the -PLGF-$2_{123\text{-}144}$ variant of each treatment with the WT variants. As shown in Figure 1, at seven days, there is a statistically significant difference between the -PlGF-$2_{123\text{-}144}$ eGF triple therapy treatment and the WT triple therapy treatment, for the response variable of wound extent. For wound area, and at three days, the different is not significant.

We turn now to the second question, regarding the cellular milieu. Using the wound sections from the previous experiments, with all twelve groups, at both three days and seven days, we measured the

percentages of each of the types of cells discussed in the preceding sections. Figures 2 through 7 show these percentages, one figure per type of cell, for each of the twelve groups. As above, we carried out ANOVA/Kruskal-Wallis tests to determine if there were differences between the treatments, and appropriate post-hoc tests to compare each treatment to the diabetic non-treatment group. For each type of cell, we also compared eGF (ECM-super-affinity) and WT GF variants of each treatment. The results are discussed in the Results section.

Using R, we investigated whether percentages of each type of cell had predictive power for wound area and wound extent. For each cell type, we fit linear models using the percentage of live cells as our explanatory variable and wound extent as the response, again using scatterplots to confirm the linear relationship. We then fit linear models with wound area as the response variable. For each model, we recorded the adjusted R^2 value and the p-value, and these are displayed in Table 1. For wound extent, the percentage of effector T cells was a statistically significant predictor, with an adjusted R^2 of 0.036. For wound area, the percentage of M2 macrophages (and the percentage with arginase) were statistically significant predictors, with R^2 of 0.08 (respectively, 0.058). We caution that the p-values in Table 1 should be interpreted with caution, as so many models were fit. However, we were cautious not to include models that were overfit to our data. We used cross-validation to detect and discard such models, including models with higher-order interaction terms, or too many explanatory variables. As the present paper is the first to consider the question of the predictive power of live cell percentages for wound healing, we hope subsequent papers will confirm the relationships we identify as significant in the current exploratory analysis.
