# Supplementary material for: VEGF-A, PDGF-BB and HB-EGF engineered for promiscuous super affinity to the extracellular matrix improve wound healing in a model of type 1 diabetes
Source: NPJ Regen Med. 2021 Nov 18;6:76. doi: 10.1038/s41536-021-00189-1 (PMC8602425; doi:10.1038/s41536-021-00189-1)
Supplement: Supplementary file 2 — Reporting summary. [file 41536_2021_189_MOESM2_ESM.pdf]

nature portfolio

Corresponding author(s): Jeffrey Hubbell

Last updated by author(s): Jun 4, 2021

Reporting Summary

Nature Portfolio wishes to improve the reproducibility of the work that we publish. This form provides structure for consistency and transparency in reporting. For further information on Nature Portfolio policies, see our Editorial Policies and the Editorial Policy Checklist.

Statistics

For all statistical analyses, confirm that the following items are present in the figure legend, table legend, main text, or Methods section.

n/a	Confirmed	
☐	☒	The exact sample size (*n*) for each experimental group/condition, given as a discrete number and unit of measurement
☐	☒	A statement on whether measurements were taken from distinct samples or whether the same sample was measured repeatedly
☐	☒	The statistical test(s) used AND whether they are one- or two-sided<br>*Only common tests should be described solely by name; describe more complex techniques in the Methods section.*
☐	☒	A description of all covariates tested
☐	☒	A description of any assumptions or corrections, such as tests of normality and adjustment for multiple comparisons
☐	☒	A full description of the statistical parameters including central tendency (e.g. means) or other basic estimates (e.g. regression coefficient) AND variation (e.g. standard deviation) or associated estimates of uncertainty (e.g. confidence intervals)
☐	☒	For null hypothesis testing, the test statistic (e.g. *F*, *t*, *r*) with confidence intervals, effect sizes, degrees of freedom and *P* value noted<br>*Give P values as exact values whenever suitable.*
☒	☐	For Bayesian analysis, information on the choice of priors and Markov chain Monte Carlo settings
☒	☐	For hierarchical and complex designs, identification of the appropriate level for tests and full reporting of outcomes
☒	☐	Estimates of effect sizes (e.g. Cohen's *d*, Pearson's *r*), indicating how they were calculated

Our web collection on statistics for biologists contains articles on many of the points above.

Software and code

Policy information about availability of computer code

Data collection	*Provide a description of all commercial, open source and custom code used to collect the data in this study, specifying the version used OR state that no software was used.*
Data analysis	Data was analyzed with PRISM versions 7 and 8, and statistics were performed by the program R

For manuscripts utilizing custom algorithms or software that are central to the research but not yet described in published literature, software must be made available to editors and reviewers. We strongly encourage code deposition in a community repository (e.g. GitHub). See the Nature Portfolio guidelines for submitting code & software for further information.

Data

Policy information about availability of data

All manuscripts must include a data availability statement. This statement should provide the following information, where applicable:

- Accession codes, unique identifiers, or web links for publicly available datasets
- A description of any restrictions on data availability
- For clinical datasets or third party data, please ensure that the statement adheres to our policy

The datasets generated during the current study are available from the corresponding author on reasonable request

Field-specific reporting

Please select the one below that is the best fit for your research. If you are not sure, read the appropriate sections before making your selection.

- [x] Life sciences
- [ ] Behavioural & social sciences
- [ ] Ecological, evolutionary & environmental sciences

For a reference copy of the document with all sections, see nature.com/documents/nr-reporting-summary-flat.pdf

Life sciences study design

All studies must disclose on these points even when the disclosure is negative.

Sample size	Since this study was exploratory, we decided a target size of 8 mice per group was sufficient, based upon previous studies (Briquez and Martino, Science, 2014)
Data exclusions	There were no data exclusions
Replication	Our study was conducted as a walk-in clinic, and as such our findings are quite robust, and are not attributable to any particular confounding variable on any given day.
Randomization	NOD mice become diabetic stochastically, and we performed our experiment as a walk-in clinic. Thus which mice became diabetic, and when, was entirely out of our control. Mice were grouped for treatment randomly after surgery.
Blinding	Since we were analyzing the results of specific treatments on wounds, blinding was not possible. It wouldn't be possible to have a blinded study that also looked at all the mouse-specific variables our study did, for instance

Reporting for specific materials, systems and methods

We require information from authors about some types of materials, experimental systems and methods used in many studies. Here, indicate whether each material, system or method listed is relevant to your study. If you are not sure if a list item applies to your research, read the appropriate section before selecting a response.

Materials & experimental systems

n/a	Involved in the study	
☒	☐	Antibodies
☒	☐	Eukaryotic cell lines
☒	☐	Palaeontology and archaeology
☐	☒	Animals and other organisms
☒	☐	Human research participants
☒	☐	Clinical data
☒	☐	Dual use research of concern

Methods

n/a	Involved in the study	
☒	☐	ChIP-seq
☐	☒	Flow cytometry
☒	☐	MRI-based neuroimaging

Animals and other organisms

Policy information about studies involving animals; ARRIVE guidelines recommended for reporting animal research

Laboratory animals	NOD mice, aged between 8 and 30 weeks old, and NOR mice 12 weeks old
Wild animals	None
Field-collected samples	None
Ethics oversight	Approved by the University of Chicago IACUC

Note that full information on the approval of the study protocol must also be provided in the manuscript.

Flow Cytometry

Plots

Confirm that:

☒ The axis labels state the marker and fluorochrome used (e.g. CD4-FITC).

☒ The axis scales are clearly visible. Include numbers along axes only for bottom left plot of group (a 'group' is an analysis of identical markers).

☒ All plots are contour plots with outliers or pseudocolor plots.

☒ A numerical value for number of cells or percentage (with statistics) is provided.

Methodology

Sample preparation	to reduce variability, all wound samples were digested and frozen in multiple aliquots. for all the data in the paper, a single large (>200 sample) flow cytometry run was performed on the same calibration and compensation, thus reducing the variability between experiments.
Instrument	BD Fortessa
Software	FACSDIVA
Cell population abundance	Cells were gated for live cells using FSC-a vs SSC-a, followed by FCS-a vs FSC-h, followed by FSC-h vs FSC-w, followed by SSC-h vs SSC-w, then followed by live-dead aqua staining. We could expect 10,000+ cells per wound aliquot
Gating strategy	We used bead intensity and compensation data to establish where to draw the gates

☒ Tick this box to confirm that a figure exemplifying the gating strategy is provided in the Supplementary Information.
